# Augmenting the Pressure-Based Pasteurization of *Listeria monocytogenes* by Synergism with Nisin and Mild Heat

**DOI:** 10.3390/ijerph17020563

**Published:** 2020-01-15

**Authors:** Sadiye Aras, Md Niamul Kabir, Shahid Chowdhury, Aliyar Cyrus Fouladkhah

**Affiliations:** 1 Public Health Microbiology Laboratory, Tennessee State University, Nashville, TN 37209, USA; saras@my.tnstate.edu (S.A.); mkabir@my.tnstate.edu (M.N.K.); schowdh1@tnstate.edu (S.C.); 2 Cooperative Extension Program, Tennessee State University, Nashville, TN 37209, USA

**Keywords:** *Listeria monocytogenes*, high-pressure processing, nisin, pressure-assisted thermal processing, synergism

## Abstract

The current study investigated *Listeria monocytogenes* inactivation using mild heat with elevated hydrostatic pressure and nisin under buffered condition. A four-strain pathogen mixture was exposed to 0 (control) and up to 9 min of (1) 4 °C elevated pressure; (2) 4 °C elevated pressure and nisin; (3) 4 °C nisin; (4) heat at 40 °C; (5) 40 °C elevated pressure; (6) 40 °C elevated pressure and nisin; and (7) 40 °C nisin. Elevated hydrostatic pressure at 400 MPa (Hub880 Explorer, Pressure BioScience Inc., Easton, MA, USA) and nisin concentration of 5000 IU/mL were used in the trials. Analyses of variance were conducted, followed by Dunnett’s- and Tukey-adjusted means separations. Under conditions of these experiments, nisin augmented (*p* < 0.05) decontamination efficacy of 40 °C heat and elevated hydrostatic pressure treatments, particularly at treatment interval of 3 min. This synergism with nisin faded away (*p* ≥ 0.05) as the treatment time for thermal, high-pressure, and thermal-assisted pressure processing increased. The results of our study, thus, exhibit that practitioners and stakeholders of pressure-based technologies could benefit from synergism of mild heat and nisin for short-term, high-pressure pasteurization treatments to achieve microbial safety and economic feasibility comparable to traditional heat-treated products.

## 1. Introduction

Considering the complexity of microbial communities, their adaptability to environmental changes, and their ubiquitous nature, contamination of water supplies and food products with pathogens of public health concern is practically unavoidable. As such, assuring safety of food and water supplies from natural and anthropogenic microbial pathogens is a daunting task and a moving target [1,2]. Among more than 200 pathogenic organisms that could cause foodborne illnesses, hospitalizations, and deaths, various serotypes of *Listeria monocytogenes* (particularly 4b, 1/2a, and 1/2b) are of great public health significance. Review of the National Outbreak Reporting System (NORS) of the Centers for Disease Control and Prevention (CDC) indicates that from 1998–2017, there have been at least 77 single or multi-state outbreaks associated with this pathogen, leading to 881, 689, and 140 cases of confirmed illness, hospitalization, and death episodes, respectively [3]. Of particular importance is around 78% and 20% hospitalization and death rates, respectively, associated with these outbreaks. Over 97% of the outbreaks were also associated with contaminated food [3]. Epidemiological evidence from the active surveillance data of the CDC also reveals similar trends with at least 1591 annual illnesses associated with *L. monocytogenes* (>99% of the cases are foodborne), with 94% hospitalization rate and 15.9% death rate in the United States [4]. Statistics from the European Food Safety Agency provide a similar epidemiological image. In 2016, five *L. monocytogenes* outbreaks were reported, consisting of 25 cases with 56% hospitalization rate [5,6]. In the same year, thirty countries of EU/EEA collectively reported 2555 confirmed listeriosis cases with the highest rate of illness for infants below one year of age and among senior citizens of >64 years of age, with an overall increasing trend in notification rates [5,6].

In addition to consumer insecurity and economic burden to public health infrastructure and the private industry due to costs associated with testing, recalls, and outbreak investigations, *L. monocytogenes* is estimated to cause 8800 disability-adjusted life years (DALYs) every year in the U.S. [7]. Besides fever and muscle pain, infections with the pathogen could cause headache, stiff neck, confusion, convulsion, and loss of balance. Additionally, during pregnancy, infections with *L. monocytogenes* could lead to fever, flu-like symptoms, and muscle pain that could lead to cases of miscarriage, premature delivery, stillbirth, or the newborn life-threatening infection [8]. The current public health burden of this pathogen indicates a need for more efficacious and adaptable methods for elimination of this pathogen, while meeting the consumer demands of the 21st century.

Nisin is a natural peptide antimicrobial agent that is produced by *Lactococcus lactis*, the first bacteriocin that was legally approved for use in an array of food products and was first commercially marketed in 1953 [9]. The ingredient is part of the generally recognized as safe list of the U.S. Food and Drug Administration [10] and has been used as an efficacious antimicrobial against an array of pathogens, particularly Gram-positive bacteria in various concentrations, such as 5000 IU per gram of the products [11]. While an array of studies [12,13,14,15,16,17] are available investigating effects of nisin on enhancing the efficacy of thermal and nonthermal pasteurizations, there are limited studies to discuss the efficacy of this antimicrobial under elevated hydrostatic pressure and under precise control of temperature and intrinsic (e.g., water activity, nutrients, pH) and other extrinsic (e.g., relative humidity, presence of gases, activity and presence of competitor microorganisms) factors. This is primarily due to the fact that precision in controlling and monitoring the temperature of the pressure-based treatments has been a major challenge for academic and industrial high-pressure research in the last several decades [18,19,20].

High-pressure pasteurization is a nonthermal processing that is gaining further popularity in food manufacturing due to its ability to preserve the fresh-like qualities of the product while eliminating the microbial pathogens and spoilage microorganisms. Manufacturers currently rely on treatments typically lasting for 3 min at intensity levels up to 87,000 pound-force per square inch (600 MPa) [20,21]. Utilization of the treatments with intensity levels of less than 600 MPa could be of great importance to the manufacturers since low-intensity treatments could minimize the cost of the operation and provide a competitive market for pressure-treated products that currently have slightly higher operational costs relative to traditional heat-treated products [20]. Our study investigates synergism of nisin with high-pressure and thermal-assisted high-pressure pasteurizations of *L. monocytogenes* to assure microbial safety and economic feasibility of the pressure-based inactivation of this microbial pathogen of public health significance.

## 2. Materials and Methods

### 2.1. L. monocytogenes Strains, Culture Preparation, and Inoculation Procedures

A four-strain mixture of pathogenic *L. monocytogenes* (ATCC 51772, 51779, BAA-2657, and13932) was utilized in the current study. Each strain was selected based on public health and food industry relevance, representing diverse lineages and ribotypes. For each individual strain, a 0.5 mL portion of glycerol frozen stock was transferred to 10 mL of Tryptic Soy Broth (Difco, Becton Dickinson, Franklin Lakes, NJ, USA) with yeast extract (0.6%) supplemented (TSB + YE). After 37 °C incubation for 24 h, a 100 µL portion of cultured suspension was subcultured aseptically (for each strain individually) in 10 mL of sterile TSB + YE for a second 24 h of incubation at 37 °C. Cells of each of the four separately subcultured strains (2 mL per strain) were subsequently harvested by centrifuge at 6000 revolutions per minute (3548 g, for 88 mm rotor) lasting 15 min (Eppendorf North America, Hauppauge, NY, USA; Model 5424, Rotor FA-45-24-11). This step was repeated twice by re-suspending the cells (each strain individually) in phosphate-buffered saline (PBS, VWR International, Radnor, PA, USA), to remove the sloughed components of cells, secondary metabolites excreted, and growth medium. The four purified strains were then composited into one inoculation cocktail, serially 10-fold diluted in PBS for achieving the target inoculation of 7.5 log CFU/mL.

### 2.2. Application of Nisin, Mild Heat, and Elevated Hydrostatic Pressure

Powdered nisin (Sigma-Aldrich, St. Louis, MO, USA), a generally recognized as safe natural antimicrobial [10], was used at a concentration of 5000 IU/mL (1000 IU = 0.025 mg nisin), as a concentration of relevance to stakeholders and the private industry [11]. It is important to note that International Unit (IU) of nisin by definition is the amount of nisin needed to inhibit a single cell of *Streptococcus agalactiae* in 1 mL of broth [22]. The powder nisin product was mixed with 2 mL PBS and centrifuged for one min at 3000 revolutions per minute (RPM) using the above-mentioned rotor and centrifuge instrument to remove insoluble solids and assure possibility for filter sterilization [23,24]. The supernatant was then filter-sterilized (0.2 µm PES Sterile Syringe Filter, VWR International, Radnor, PA, USA) and used at concentration of 5000 IU/mL in PBS inoculated with the above-mentioned pathogen cocktail. At two temperatures of 4 and 40 °C and at an elevated hydrostatic pressure of 400 MPa (Pressure BioScience Inc., Hub880 Explorer unit), the inoculated PBS samples were exposed to pressure, mild heat, nisin, and their combinations. Temperature values of the experiments were controlled by a water jacket made from stainless steel, covering the treatment chamber. The jacket was mechanically linked to a refrigerated circulating water bath (VWR International, Radnor, PA, USA, Model refrigerated 1160s), and temperature values were recorded by a t-type thermocouple (Omega Engineering Inc., Norwalk, CT, USA) as further detailed in our open access recent publications [20,21,25]. Pressure treatments occurred inside the PULSE tubes without disk (Pressure BioScience Inc., Easton, MA, USA) that contained 1.5 mL of inoculated PBS with or without presence of the antimicrobial. Treatments were applied for 0, 3, 6, and 9 min, with recording and monitoring of temperature and treatment parameters in every 3 s using the HUB Explorer PBI software Version 1.0.8 (Pressure BioScience Inc., Easton, MA, USA). In total, seven treatments were investigated: (1) 4 °C elevated pressure; (2) 4 °C elevated pressure and nisin; (3) 4 °C nisin; (4) heat at 40 °C; (5) 40 °C elevated pressure; (6) 40 °C elevated pressure and nisin; and (7) 40 °C nisin. Although thermal-assisted, high-pressure treatments could utilize temperatures as high as 90 °C and above [26], temperatures higher than 50 °C could disqualify a product from the terminology “cold-pressed” or “high-pressure-processed,” one of the main advantages for marketing of the pressure-treated products, and additionally could limit the efficacy of bacteriocins [27]. Thus, selection of temperature for an efficacious and feasible thermal-assisted high-pressure treatment would need to consider the scientific literature and national and international regulatory requirements for pressure-based pasteurization.

### 2.3. Neutralization, and Microbiological and pH Analyses

Before microbiological analyses, samples were neutralized by 3 mL aliquots of D/E neutralizing broth (Difco, Becton Dickinson, Franklin Lakes, NJ USA) for each milliliter of treated samples for assuring precision of antimicrobial (nisin) exposure time. Samples were additionally placed on ice–water slurry immediately after the treatments for precise exposure time of 40 °C samples. Each treated sample was subsequently diluted 10-fold serially in Maximum Recovery Diluent (Difco, Becton Dickinson, Franklin Lakes, NJ, USA) to assure maximizing the recovery of the viable but injured microbial cells. Then, the neutralized diluents were spread-plated on PALCAM base agar medium (Becton, Dickinson and Company, Sparks, MD, USA) that was supplemented with Ceftazidime (Becton, Dickinson and Company, Sparks, MD, USA) for selective growth of *L. monocytogenes*, and on the surface of Tryptic Soy Agar supplemented with yeast extract (TSA + YE) for nonselective enumeration. Both selective and nonselective media were then incubated for 48 h at 37 °C. Microbial colonies were then physically counted and log-transformed. The pH of each treated sample was measured after treatment and after neutralization by a pH meter calibrated at pH = 4.00, pH = 7.01, and pH = 10.01 (Mettler Toledo, Columbus, OH, USA).

### 2.4. Experimental Design and Descriptive and Inferential Statistics

Two blocks, each containing sample size of three repetitions per analysis, were used, on the basis of previously conducted power analyses in Public Health Microbiology laboratory by *Proc Power* of SAS statistical package (SAS Institute, Cary, NC, USA) [28]. Each block of the study was a biologically independent trial, thus the experiment was a complete randomized block design. Each of the above-mentioned repetitions per block was also repeated in duplicate as microbiological/instrumental replicates. Hence, every reported value (mean ± standard deviation) is the average of 12 independent observations (two blocks, three repetitions, two microbiological/instrumental replications). Data management, log conversions, and descriptive exhibitions were conducted using Microsoft Excel. The data were then imported into SAS (SAS Institute, Cary, NC, USA), and each inferential statistical analysis carried out at 5% type 1 error (α = 0.05). Analysis of variance (ANOVA) was then performed using the *Proc GLM* of SAS followed by two mean separation methods. Pairwise comparisons were accomplished by a Tukey-adjusted test for all treated samples and controls, the outcome of these analyses are incorporated in graphs using uppercase alphabet(s). The treated samples and the untreated controls were additionally compared using a Dunnett’s-adjusted mean separation; those treatments statistically different (*p* < 0.05) than the control are marked with * sign, based on Dunnett’s-adjusted analyses. Microsoft Excel and version 1.7 of GInaFiT [29,30] software (Katholieke Universitiet, Leuven, Belgium) were used to calculate inactivation indices (D- and K_max_ values).

## 3. Results and Discussion

Intrinsic factors of a sample play critical roles on validity of a microbiological hurdle validation study. As further delineated in Section 2.3., the pH of the samples was measured after each treatment (and for nontreated controls). The samples were then neutralized by D/E neutralizing broth before each microbiological analysis. Prior to neutralization, the pH values were similar (*p* ≥ 0.05) and were on average 7.14 ± 0.2 (range 6.96 to 7.43). Counts after neutralization were also similar among the samples, ranging from 7.00 to 7.82 (average of 7.64 ± 0.1). The standard deviations of both neutralized and non-neutralized samples were ranging from 0.0 to 0.3. In addition to pH, temperature is another critical factor associated with pressure-based pasteurization validation studies [20]. In the current study, as discussed in the Section 2.2., the trials were conducted under precise control of temperature using a chamber that is covered by a steel jacket, mechanically connected to a circulating water bath with refrigeration. For high-pressure, high-pressure and nisin, and only nisin-treated samples at 4 °C and for mild heat (e.g., 40 °C), high-pressure with mild heat, high-pressure and nisin with mild heat, and nisin with mild heat samples, the temperature values were similar before and after treatments. For 4 °C samples, the temperature measurements were 5.4 ± 0.2 (ranging from 5.1 to 5.6) and 5.4 ± 0.2 (ranging from 4.9 to 5.6) before and after treatments, respectively. Samples treated under mild heat (40 °C) had temperature measurements of 39.4 ± 0.6 (ranging from 38.1 to 40.3) and 39.7 ± 0.6 (ranging from 38.5 to 40.4) before and after treatments, as well.

It is important to note that *L. monocytogenes* is a relatively diverse pathogen with a genome size that could range from 2.94 to 3.11 Mbp [31]. Other studies have also reported a similar range of genome for the bacterium, such as sizes ranging from 2.95 to 3.11 Mbp [32]. In addition, microbial pathogens could carry extrachromosomal material that could alter their capability in responding to environmental stressors. Thus, selection of strains is also a crucial part of a microbiological challenge study. One strain used in this study (ATCC 13932) is isolated from spinal fluid of a child with meningitis due to *L. monocytogenes* infection. Another strain (ATCC BAA-2657) is a strain that was provided to American Type Culture Collection from the U.S. Centers for Disease Control and Prevention. The other two strains were isolated from food companies (ATCC 51772 and ATCC 51779). Thus, the selected strains provide generalizable outcome of public health and food industry significance. Understandably, there are unknown number of *L. monocytogenes* strains and some inherently might exhibit more or less susceptibility to environmental and processing stressors relative to the selected strains in this study. Choosing strains with rare phenotypic characteristics may yield results that are not typical, practical, or realistic and might lead to recommendations that are overly conservative or overly liberal for inactivation of this pathogen. This complexity has to be taken into considerations prior to adaption of microbiological validation studies to assure an operation is safeguarding the public health, minimally affecting the organoleptic, structural, and nutritional quality of a product, and to assure that a process is economically feasible and meeting the regulatory requirements for commerce. Stakeholders could additionally benefit from a multiple-hurdle technology approach to assure cost-effectiveness and feasible efficacy of an operation [33].

### 3.1. Inactivation of L. monocytogenes by High-Pressure Pasteurization

**Figure 1:**
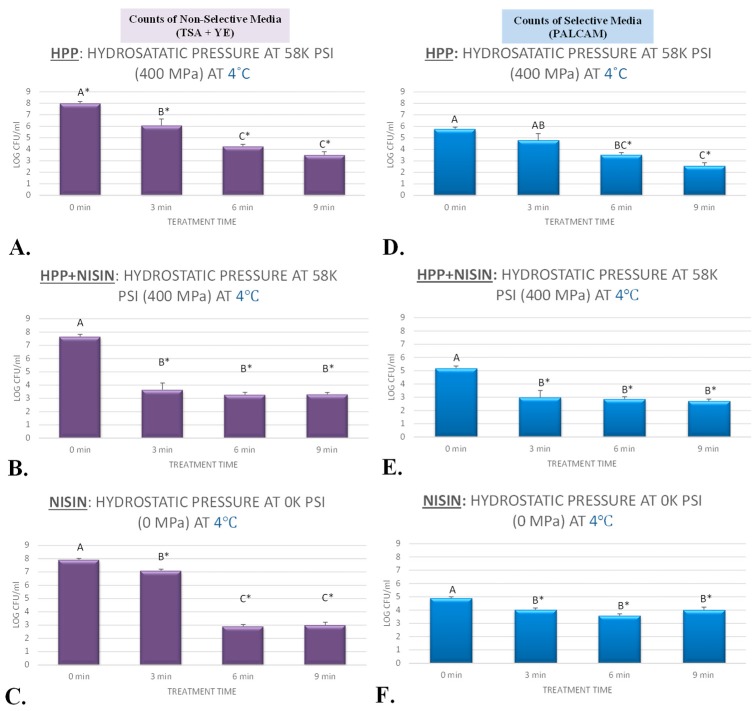
Inactivation of four-strain cocktail of *L. monocytogenes* (ATCC^@^ 51772, 51779, BAA-2657, and 13932) in sterilized phosphate-buffered saline, treated by nisin (5000 IU/mL) and elevated hydrostatic pressure at 400 MPa (Barocycler Hub880 Explore, Pressure Bioscience Inc., Easton, MA, USA) for 0, 3, 6, and 9 min at 4 °C. In each graph and for each microbiological medium (PALCAM (selective counts), TSA + YE (nonselective counts)) separately, columns of each time interval followed by different uppercase letters are representing log CFU/mL values (Mean ± SD) that are statistically (*p* < 0.05) different (Tukey-adjusted ANOVA). Uppercase letters followed by * sign are statistically (*p* < 0.05) different than untreated control (not treated with antimicrobial) (Dunnett’s-adjusted ANOVA). (**A**) Nonselective counts (Tryptic Soy Agar + yeast extract [TSA + YE]), treated by hydrostatic pressure at 400 MPa (58 K pound-force per square inch [PSI]) at 4 °C; (**B**) Nonselective counts (TSA + YE), treated by hydrostatic pressure at 400 MPa (58 K PSI) and exposed to nisin (5000 IU/mL) at 4°C; (**C**) nonselective counts (TSA + YE), only exposed to nisin (5000 IU/mL) at 4 °C. (**D**) Selective counts (PALCAM), treated by hydrostatic pressure at 400 MPa (58 K PSI) at 4 °C; (**E**) Selective counts (PALCAM), treated by hydrostatic pressure at 400 MPa (58 K PSI) and exposed to nisin (5000 IU/mL) at 4°C; (**F**) Selective counts (PALCAM), only exposed to nisin (5000 IU/mL) at 4 °C.

**Figure 2:**
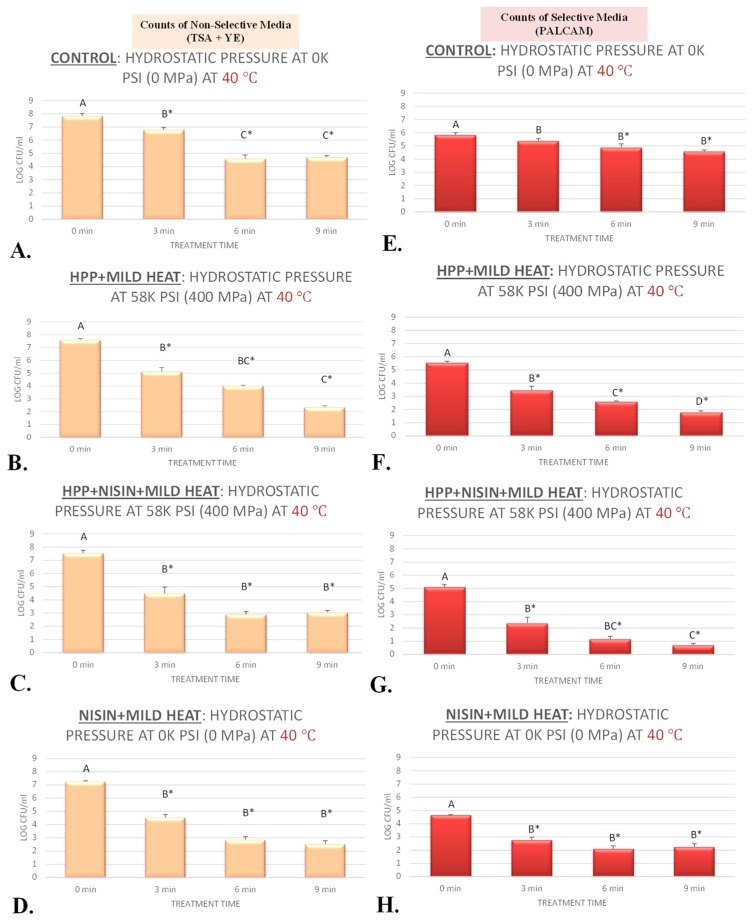
Inactivation of four-strain cocktail of *L. monocytogenes* (ATCC^@^ 51772, 51779, BAA-2657, and 13932) in sterilized phosphate-buffered saline, treated by nisin (5000 IU/mL) and elevated hydrostatic pressure at 400 MPa (Barocycler Hub880 Explore, Pressure Bioscience Inc., Easton, MA, USA) for 0, 3, 6, and 9 min at 40 °C. In each graph and for each microbiological medium (PALCAM (selective counts), TSA + YE (non-selective counts)) separately, columns of each time interval followed by different uppercase letters are representing log CFU/mL values (Mean ± SD) that are statistically (*p* < 0.05) different (Tukey-adjusted ANOVA). Uppercase letters followed by * sign are statistically (*p* < 0.05) different than untreated control (not treated with the antimicrobial) (Dunnett’s-adjusted ANOVA). (**A**) Nonselective counts (TSA + YE), treated by heat at 40 °C; (**B**) Nonselective counts (TSA + YE), treated by hydrostatic pressure at 400 MPa (58 K PSI) and at 40 °C; (**C**) Nonselective counts (TSA + YE), treated by hydrostatic pressure at 400 MPa (58 K PSI) and exposed to nisin (5000 IU/mL) at 40 °C; (**D**) Nonselective counts (TSA + YE), exposed to nisin (5000 IU/mL) at 40 °C. (**E**) Selective counts (PALCAM), treated by heat at 40 °C; (**F**) Selective counts (PALCAM), treated by hydrostatic pressure at 400 MPa (58 K PSI) and at 40 °C; (**G**) Selective counts (PALCAM), treated by hydrostatic pressure at 400 MPa (58 K PSI) and exposed to nisin (5000 IU/mL) at 40 °C; (**H**) Selective counts (PALCAM), exposed to nisin (5000 IU/mL) at 40 °C.

The current study evaluated sensitivity of *L. monocytogenes* to elevated hydrostatic pressure at 400 MPa (58 K PSI) and at target temperatures of 4 and 40 °C (Figure 1A,D and Figure 2B,F) for up to 9 min. The microbial counts were enumerated from selective media (PALCAM counts) as well as nonselective media (TSA) with yeast extract supplementation (TSA + YE counts). PALCAM counts were consistently lower (*p* < 0.05) than those obtained from TSA + YE for the vast majority of time/treatment combinations, which could be attributed to presence of selective and differential agents and pH indicator(s) with bactericidal and/or bacteriostatic properties in selective media. This has been consistent with previous literature and is attributed to presence of selective and differential agents in PALCAM that limits the recovery of injured but viable cells [34,35]. Addition of yeast extract to TSA was based on preliminary experiments and to enhance recovery of viable but injured cells after each treatment [20]. The PALCAM and TSA + YE counts before treatment (untreated control) were 5.77 ± 0.2 and 7.98 ± 0.1 log CFU/mL (mean ± standard deviation), respectively, at 4 °C (Figure 1A,D). These counts were reduced to 4.80 ± 0.6 (PALCAM counts) and 6.07 ± 0.6 (TSA + YE counts) after a 3 min treatment at 400 MPa and at 4 °C (Figure 1A,D). The log reductions obtained from TSA + YE counts were predictably more pronounced for treatments with longer treatment times, and were 3.7 and 4.5 for samples treated for 6 and 9 min at the above-mentioned level of pressure (Figure 1A,D). Mild temperature augmented the sensitivity of the pathogen to elevated hydrostatic pressure. The log reduction values (TSA + YE counts) associated with 3, 6, and 9 min treated samples at 400 MPa and at 40 °C were 2.5, 3.6, and 5.2, respectively (Figure 2B,F). This enhancement in decontamination effectiveness of a high-pressure pasteurization unit in the presence of mild heat is similar to that in recent literature, where *L. monocytogenes* were reduced more effectively via thermal-assisted pressure pasteurization relative to treatments of elevated hydrostatic pressure at 4 °C [25]. Results of the current study are also in harmony with the previous literature, where it has been shown that a pressure treatment at temperatures around 43 °C could lead to reductions of >5 log CFU for *L. monocytogenes* [18]. It is noteworthy that many of the existing studies in the literature were conducted with equipment that has limited capability to control and monitor the temperature [18], and the current study was an endeavor to determine the sensitivity of the pathogen to pressure, heat, and nisin by precisely controlling the temperature and all intrinsic and extrinsic factors in buffered environment.

### 3.2. Inactivation of L. monocytogenes by Nisin

As an antimicrobial that is generally recognized as safe (GRAS), nisin is a polypeptide bacteriocin that could be efficacious against an array of foodborne pathogens and spoilage organisms [36]. Under conditions of this experiment, we exhibited up to 5 log CFU/mL *L. monocytogenes* reductions associated with application of nisin alone. Specifically, utilizing the above-referenced nonselective media, the pathogen count before treatment was 7.92 ± 0.0 log CFU/mL (mean ± standard deviation) at 4 °C (Figure 1C). These nonselective counts were statistically reduced (*p* < 0.05) to 7.09 ± 0.1, 2.91 ± 0.1, and 3.00 ± 0.1 after 3, 6, and 9 min of exposures to 5000 IU/mL nisin at 4 °C, respectively (Figure 1C). Although efficacious compared with control (*p* < 0.05), 3 min and 6 min treatments at 4 °C were not statistically (*p* ≥ 0.05) different from each other (Figure 1C). Log reductions associated with nonselective counts were 0.8, 5.0, and 4.9 log CFU/mL for samples treated with 5000 IU/mL nisin for 3, 6, and 9 min at 4 °C, respectively (Figure 1C). Counts obtained from selective medium (PALCAM counts) at 4 °C also revealed similar trends, although as it was discussed in Section 3.1., PALCAM counts were consistently lower than the nonselective counts. Under the condition of our experiment, nisin (5000 IU/mL) at the higher temperature of 40 °C were also capable of significantly (*p* < 0.05) reducing the pathogen counts. Elevated temperature did not augment (*p* ≥ 0.05) the efficacy of the antimicrobial relative to 4 °C (Figure 2D,H) for treatments longer than 5 min, while it augmented (*p* < 0.05) the efficacy of the treatment for 3 min treated samples (Figure 1C,F and Figure 2D,H). The *L. monocytogenes* log reductions (*p* < 0.05), obtained from nonselective media, were 0.8 and 2.7 log CFU/mL for samples exposed to nisin (5000 IU/mL) for 3 min at 4 and 40 °C. These reductions were similar (*p* ≥ 0.05) and were 5.0 and 4.4 log CFU/mL for samples exposed to nisin (5000 IU/mL) for 6 min, and were 4.9 and 4.7 for 9 min treated samples, at 4 and 40 °C (Figure 1C,F and Figure 2D,H).

Efficacy of nisin for decontamination of this Gram-positive microbial pathogen is in harmony with previous studies where the application of this antimicrobial had been efficacious for up to 5-log reduction of *L. monocytogenes* [36,37]. Similarly, addition of nisin had been previously reported to increase sensitivity of *L. monocytogenes* to elevated hydrostatic pressure [18], where a 345 MPa treatment at 25 °C lasting for 10 min had been augmented in the presence of 3000 IU/mL of nisin [38]. The current study investigated the synergism of nisin and pressure-based pasteurization against the planktonic cells of *L. monocytogenes*. Previous studies have also indicated that pressure at an intensity level of 400 MPa lasting for up to 10 min in the presence of 1000 IU/mL nisin could also be efficacious for the treatment of sessile cells of the pathogen when tested against *L. monocytogenes* biofilm [39]. Nisin was also similarly efficacious against *Listeria innocua* at 35 and 50 °C and pressure intensity of 200–500 MPa [40]. In addition to antilisterial activities, this naturally occurring antimicrobial has been tested for enhancing the pressure-based pasteurization of bacterial spores [41], *Escherichia coli* and *Staphylococcus aureus* [42], *Salmonella enterica* serovar Typhimurium and *Bacillus subtilis* [43], and pathogenic *Cronobacter* spp. [44].

It is crucial to note that that although the direct comparison of two studies conducted in different vehicles is inherently unfeasible since each medium or product has different intrinsic and extrinsic factor(s), comparing various studies using similar settings could provide trends and a medium for evidence-based decision-making by stakeholders, an opportunity for future academic and industrial researchers to assimilate existing knowledge and formulate new experiments, and create an accelerated opportunity for risk assessment by public health practitioners. As delineated earlier in Section 3, this study was conducted in PBS to eliminate intrinsic and extrinsic factors associated with specific food products to study sensitivity of the pathogen to elevated pressure, nisin, and mild heat. Complex food vehicles have additional intrinsic and extrinsic factors, and specific product–pathogen interactions would need to be considered for a new product prior to adaption of results of validation studies.

### 3.3. Synergism of Nisin, Mild Heat, and Elevated Hydrostatic Pressure

Treatments of hydrostatic pressure lasting for 3 min are of particular importance since currently the private industry utilizes 3-min treatments for the vast majority of commercially available products [19,20,25]. Under the condition of our experiment at 4 °C, exposure to 400 MPa of high-pressure treatments for 3 min resulted in 1.9 log CFU/mL reductions (*p* < 0.05) of *L. monocytogenes* (Figure 1A,D). In the presence of nisin (5000 IU/mL), the same treatment at 4 °C resulted in 4.0 log CFU/mL reductions (*p* < 0.05) of the pathogen (Figure 1B,E). This exhibits the capability of nisin to augment efficacy of a high-pressure pasteurizer. While exposure to heat alone (40 °C for 3 min) resulted in a modest reduction of (*p* < 0.05) 1.1 log CFU/mL of *L. monocytogenes* (Figure 2A,E), exposure to mild heat and nisin (5000 IU of nisin at 40 °C for 3 min) resulted in reductions of (*p* < 0.05) 2.7 log CFU/mL of *L. monocytogenes* (Figure 2D,H). Similarly, exposure to mild heat and nisin and hydrostatic pressure (5000 IU of nisin at 40 °C for 3 min under 400 MPa of hydrostatic pressure) led to reductions of (*p* < 0.05) 3.0 log CFU/mL of *L. monocytogenes* (Figure 2C,G). Thus, it could be concluded that nisin could enhance the decontamination effectiveness of heat as well as pressure-based pasteurization of *L. monocytogenes* at a time interval that is relevant to the private industry. Although heat and nisin were able to enhance the efficacy of pressure-based decontamination of *L. monocytogenes*, utilization of heat and nisin simultaneously did not exhibit additional pathogen reduction effectiveness for the pressure-based pasteurization. For exposure times higher than 3 min, this synergism among pressure, heat, and nisin was less pronounced for inactivation of *L. monocytogenes*. As an example, treatment with heat (40 °C) alone for 9 min led to reductions of (*p* < 0.05) 3.1 log CFU/mL of *L. monocytogenes* (Figure 2A,E), treatment with heat and nisin (5000 IU of nisin at 40 °C for 9 min) led to reductions of (*p* < 0.05) 4.7 log CFU/mL of *L. monocytogenes* (Figure 2D,H), treatment under hydrostatic pressure and nisin (5000 IU of nisin under 400 MPa at 4 °C for 9 min) led to reductions of (*p* < 0.05) 4.3 log CFU/mL of *L. monocytogenes* (Figure 1B), and heat treatment under hydrostatic pressure and nisin (5000 IU of nisin under 400 MPa at 40 °C for 9 min) resulted in reductions of (*p* < 0.05) 4.5 log CFU/mL of *L. monocytogenes* (Figure 2C,G). It is noteworthy that the microbial counts discussed in this section (Section 3.3.) are those derived from nonselective media with yeast extract supplementation that has superior performance relative to selective media for enhancing the recovery of injured cell and thus have higher external validity [20].

### 3.4. Inactivation Indices for Inactivation of L. monocytogenes

Inactivation indices summarize the decontamination efficacy of a treatment against a specific pathogen and at specific intrinsic and extrinsic factors of the product. In our study, we calculated D-values based on the best-fitted linear model as well as K_max_ values based on the nonlinear model with highest goodness-of-fit statistics (highest R^2^). The reported D-values presented in the current study correspond to the time (in min) required to achieve a one-log reduction (i.e., 90% reduction) of *L. monocytogenes* mixture under the conditions of the study, while K_max_ exhibits the number of log cycles of reduction of *L. monocytogenes* mixture in 1/min unit.

**Figure 3:**
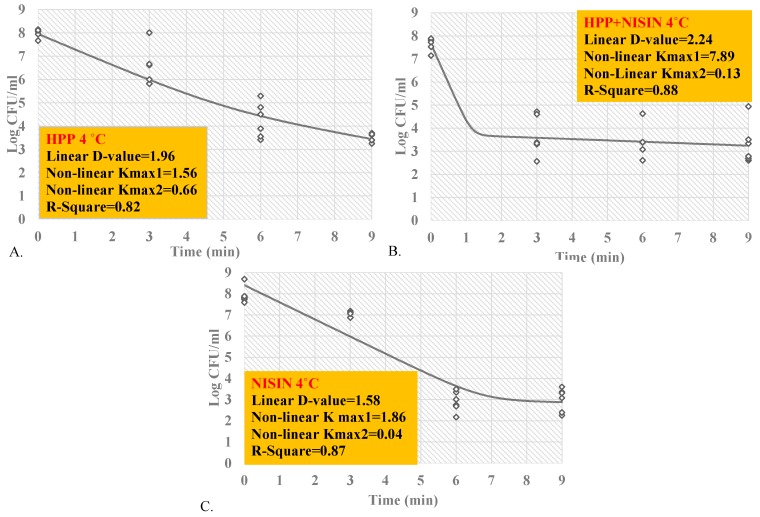
Linear and nonlinear inactivation indices for four-strain cocktail of *L. monocytogenes* (ATCC^@^ 51772, 51779, BAA-2657, and 13932) in sterilized phosphate-buffered saline, treated by nisin (5000 IU/mL) and/or elevated hydrostatic pressure at 400 MPa (Barocycler Hub880 Explore, Pressure Bioscience Inc., Easton, MA, USA) at 4 °C. (**A**) Treated by hydrostatic pressure at 400 MPa (58 K PSI) at 4 °C; (**B**) Treated by hydrostatic pressure at 400 MPa (58 K PSI) and exposed to nisin (5000 IU/mL) at 4 °C; (**C**) Exposed to nisin (5000 IU/mL) at 4 °C.

**Figure 4:**
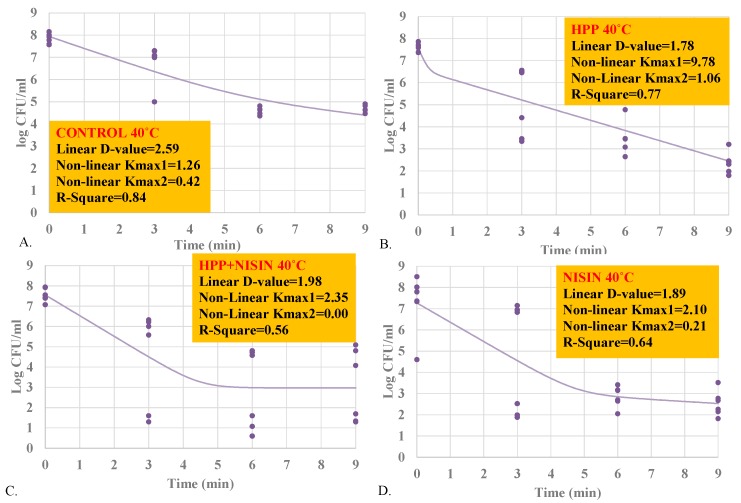
Linear and nonlinear inactivation indices for four-strain cocktail of *L. monocytogenes* (ATCC^@^ 51772, 51779, BAA-2657, and 13932) in sterilized phosphate-buffered saline, treated by nisin (5000 IU/mL) and/or elevated hydrostatic pressure at 400 MPa (Barocycler Hub880 Explore, Pressure Bioscience Inc., Easton, MA, USA) at 40 °C. (**A**) Treated by heat at 40 °C; (**B**) Treated by hydrostatic pressure at 400 MPa (58 K PSI) and at 40 °C; (**C**) Treated by hydrostatic pressure at 400 MPa (58 K PSI) and exposed to nisin (5000 IU/mL) at 40 °C; (**D**) Exposed to nisin (5000 IU/mL) at 40 °C.

At 4 °C, the D-value and inactivation K_max_ for samples treated at 400 MPa for up to 9 min were 1.96 and 1.56, respectively (Figure 3A). This indicates, with assumption of linearity, that every 1.96 min, the treatment is capable of reducing 90% of the inoculated pathogen under the intrinsic and extrinsic conditions of the experiment. Similarly, the above-mentioned treatment in the presence of 5000 IU nisin resulted in D-value and inactivation K_max_ of 2.24 and 7.89, respectively (Figure 3B), while exposure to nisin alone yielded D-value and inactivation K_max_ of 1.58 and 1.86, respectively (Figure 3C). These findings are in harmony with results of microbial log reductions in Section 3.1, Section 3.2 and Section 3.3, where nisin was efficacious to enhance the decontamination effectiveness of the pressure- and heat-based treatments at a time interval of 3 min but had its efficacy diminished at longer time intervals. At 40 °C, the D-values for heat-treated (40 °C), pressure-treated (400 MPa), pressure-treated with nisin (400 MPa, 5000 IU/mL), and nisin-treated (5000 IU/mL) samples were 2.59, 1.78, 1.98, and 1.89 min, respectively (Figure 4A–D). The inactivation K_max_ of the treatments were 1.26, 9.78, 2.35, and 2.10 (Figure 4A–D). Both linear and nonlinear inactivation indices could provide adaptable information for stakeholders’ assimilation, however, it is important to note that inactivation indices would need to be utilized and adapted with caution after studying the pathogen–product specific relationship to assure the most generalizable linear or nonlinear model had been selected [20,21,22].

## 4. Conclusions

Under conditions of the conducted trials, it was exhibited that nisin could enhance the decontamination efficacy of mild heat as well as elevated hydrostatic pressure treatments at 400 MPa. This synergistic effect was more pronounced and appreciable at a treatment time of 3 min, the exposure time that is currently the most common in private food manufacturing. This synergistic effect with nisin faded away as the treatment time for thermal, high-pressure, and thermal-assisted pressure processing increased. Thus, this synergism could be of importance for medium- or high-intensity and short-time treatments. Results of our study also exhibit that practitioners and stakeholders of pressure-based technologies could benefit from synergism of mild heat and nisin with mild pressure for short-term treatments to achieve up to >5 log reductions of *L. monocytogenes*. This could be of particular importance for achieving microbial safety and economic feasibility comparable to traditional heat-treated products and meeting regulatory requirements such as Hazard Analysis and Critical Control Point, and Food Safety Modernization Act.
